# Complementing Testicular Immune Regulation: The Relationship between Sertoli Cells, Complement, and the Immune Response

**DOI:** 10.3390/ijms24043371

**Published:** 2023-02-08

**Authors:** Rachel L. Washburn, Jannette M. Dufour

**Affiliations:** 1 Immunology and Infectious Diseases, Graduate School of Biomedical Sciences, Texas Tech University Health Sciences Center, Lubbock, TX 79424, USA; 2 Department of Cell Biology and Biochemistry, School of Medicine, Texas Tech University Health Sciences Center, Lubbock, TX 79424, USA

**Keywords:** Sertoli cells, complement, testicular immune privilege, immune regulation

## Abstract

Sertoli cells within the testis are instrumental in providing an environment for spermatogenesis and protecting the developing germ cells from detrimental immune responses which could affect fertility. Though these immune responses consist of many immune processes, this review focuses on the understudied complement system. Complement consists of 50+ proteins including regulatory proteins, immune receptors, and a cascade of proteolytic cleavages resulting in target cell destruction. In the testis, Sertoli cells protect the germ cells from autoimmune destruction by creating an immunoregulatory environment. Most studies on Sertoli cells and complement have been conducted in transplantation models, which are effective in studying immune regulation during robust rejection responses. In grafts, Sertoli cells survive activated complement, have decreased deposition of complement fragments, and express many complement inhibitors. Moreover, the grafts have delayed infiltration of immune cells and contain increased infiltration of immunosuppressive regulatory T cells as compared to rejecting grafts. Additionally, anti-sperm antibodies and lymphocyte infiltration have been detected in up to 50% and 30% of infertile testes, respectively. This review seeks to provide an updated overview of the complement system, describe its relationship with immune cells, and explain how Sertoli cells may regulate complement in immunoprotection. Identifying the mechanism Sertoli cells use to protect themselves and germ cells against complement and immune destruction is relevant for male reproduction, autoimmunity, and transplantation.

## 1. Introduction

The testis is unique as it is one of only a few immune-privileged sites within the body. Since advanced male germ cells (spermatocytes, spermatids, and spermatozoa) do not emerge until puberty, these cells express immunogenic antigens and are at risk of autoimmune destruction. Sertoli cells are important contributors to germ cell protection from autoimmune destruction, and since infertility affects about 2.5–12% of males worldwide [1], the majority of men have viable germ cells due to the development and maintenance of an immune-privileged environment in the testis.

Understanding the mechanisms behind immune responses and immune protection in reproduction is a relevant issue. The immune response is complicated and consists of many components including serum proteins, complement, antibodies, and immune cells. Of these, the complement system has been particularly understudied and underappreciated. As various complement-related proteins are expressed in the testis and have important roles in fertilization and reproduction, this review strives to provide a thorough review of the complement system, complement modulation of lymphocytes, and Sertoli cell regulation of complement in the immune-privileged environment.

## 2. Sertoli Cell Immune Regulation in the Testis

**Figure 1:**
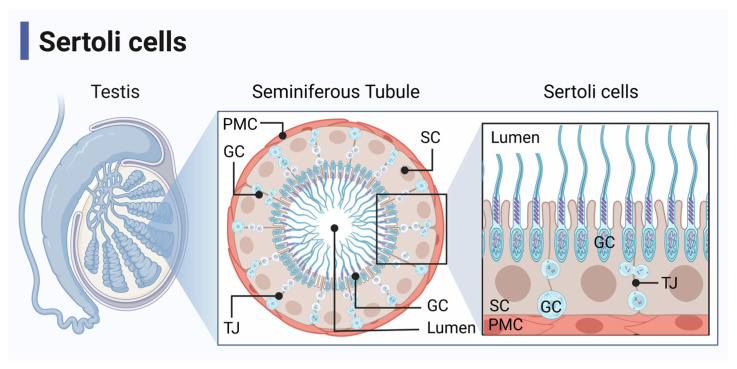
Seminiferous tubule anatomy. Sertoli cells (SC) and germ cells (GC) are located in the seminiferous tubules of the testis, which are surrounded by peritubular myoid cells (PMC). SC function to nurture spermatogenesis and protect GC from immune responses. They make up the blood–testis barrier by forming tight junctions (TJ) with adjacent SC. Figure created with BioRender accessed on 1 December 2022.

Sertoli cells are immunoregulatory cells that line the seminiferous tubules of the testis, which physiologically function to nurture spermatogenesis and protect maturing germ cells from an immune response (Figure 1) [2,3]. This immunoprotection is required due to the immunogenicity of the developing sperm since the male immune system develops central self-tolerance before the germ cells enter meiosis [4]. Sertoli cells regulate the immune response to create this immune protective environment by maintaining the blood–testis barrier and through the expression of immunoregulatory factors [2,5].

The blood–testis barrier is a physical barrier formed by tight junctions between adjacent Sertoli cells that sequester the meiotic and haploid germ cells from the immune system [2,6]. In this manner, the blood–testis barrier acts as a physical shield, directly protecting maturing sperm from destruction. However, this sequestration is incomplete since immune cells can encounter germ cell antigens as the germ cell egress into the interstitium, which influences the development of systemic tolerance [7,8]. Furthermore, since allografts and xenografts transplanted into the interstitial space, outside of the blood–testis barrier enjoy prolonged graft survival, the blood–testis barrier is not the sole factor responsible for testicular immune protection [2]. Additionally, Sertoli cells survive long-term as allografts and xenografts without the use of immune suppressants and Sertoli cell allografts have been shown to protect ectopically transplanted pancreatic islet grafts, also indicating there is more to Sertoli cell immune regulation [3,9,10,11,12].

Sertoli cells produce and secrete many different factors that can regulate immune response such as transforming growth factor beta (TGF-β), activin A, fas ligand, programmed death-ligand 1 (PD-L1), galectin-1, thrombospondin-1 (THBS1), and indoleamine-2, 3-dioxygenase (IDO) ([13,14,15,16,17,18,19,20], extensively reviewed in [21]). These factors can suppress cytotoxic immune functions to protect germ cells and have been implicated in reducing effector immune cell proliferation, inhibiting inflammatory responses, and generating regulatory immune cells [7,9,14,16,22]. Sertoli cells have also been shown to express many membrane-bound and soluble complement inhibitors that may aid in the protection of germ cells from complement activation, amplification, and destruction [23,24].

These immunoregulatory factors are most likely responsible for the extended survival of Sertoli cell allografts and xenografts, which risk immune destruction, similar to immunogenic germ cells. We have previously shown that pig Sertoli cell xenografts transplanted into immunocompetent Lewis rats survived throughout the 90-day experiment while pig islet xenografts were rejected around day six post-transplantation [9,16,25]. These Sertoli cell grafts contain an increased ratio of infiltrating regulatory T cells (Tregs) and decreased complement activation [16,25], indicating the establishment of an immunoregulatory environment within the graft that may be similar to the immune-privileged environment seen in the testis.

When testicular immune protection is disrupted, as in the case of autoimmune orchitis, immune responses are generated against the immunogenic germ cells. A hallmark of autoimmune orchitis and testicular inflammatory lesions in humans is immune cell infiltrate governed by T cells [3]. Subsequently, a humoral response can be induced, although the appearance of autoantibodies against germ cells (anti-sperm antibodies, ASA) is likely confined to epididymal immune pathologies [26,27,28]. This highlights the importance of proper complement regulation within the delicate immune privileged environment of the testis and this review will focus on complement, particularly in male reproduction.

## 3. The Complement System

As complement is understudied, this review will provide an in-depth overview of complement, complement regulation, and complement modulation of immune cells and how it affects/may affect reproduction.

### 3.1. The Cascade of Complement Activation

**Figure 2:**
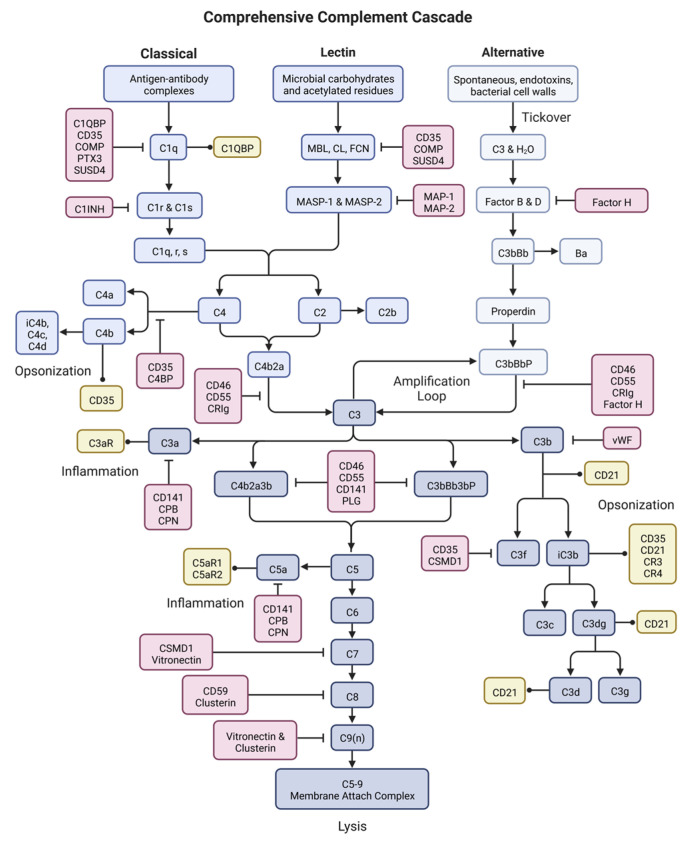
The complement system. Complement is an enzymatic cascade consisting of over 50 proteins including complement components (blues), inhibitors (reds), and receptors (yellows). Complement functions to destroy pathogens or other target cells through cell lysis, inflammation, and immune activation. This figure was created with BioRender accessed on 1 December 2022.

The complement system is a series of proteins that, when activated, undergo a cascade of proteolytic cleavages eventually leading to cell lysis, opsonization and phagocytosis, inflammation and leukocyte recruitment, and further modulation of immune responses (Figure 2) [29]. The complement cascade can be divided into three overarching events: activation, response expansion, and termination.

#### 3.1.1. Activation

Complement is activated through three primary pathways: the classical pathway, the lectin pathway, and the alternative pathway. Classical pathway activation is predominantly triggered by antigen-antibody complexes, usually involving IgG or IgM antibodies [29]. Complement protein C1q, the recognition portion of the C1 complex, binds to the antigen-antibody complex [29,30]. This binding causes a conformational shift in C1q structure, uncovering the binding site for three C1r components [31,32]. Upon C1r binding, a further conformational shift occurs allowing for binding of three C1s components, which is the enzymatic portion of the C1 complex [32]. Binding of C1s to the completed C1 complex exposes the enzymatic site and allows for cleavage of C2 and C4 to C2a/C2b and C4a/C4b, respectively [32,33]. C4a and C2b are both soluble proteins whose functions are poorly understood [34]. C4b and C2a covalently bind to each other and to the target cell to form the C3 convertase, an enzymatic complex that is the convergence point of the classical and lectin pathways [29]. C4b and C2a also act as opsonization molecules to encourage phagocytosis by cells such as macrophages and dendritic cells [35].

The lectin pathway is activated when acetylated residues and/or carbohydrate moieties on microbes, usually on bacterial and fungal surfaces, bind to the antigen-recognition molecules of the lectin pathway: mannose-binding lectins (MBL), collectins (CL), or ficolins (FCN) [29,32]. Once antigen-bound, these complexes bind to MBL-associated serine proteases 1 and 2 (MASP-1 and MASP-2), allowing them to enzymatically cleave C4 and C2 to form the C3 convertase C4bC2a [29,32]. This is the same C3 convertase that is formed by the classical pathway of activation, and the subsequent cascade steps are the same.

The alternative pathway is distinct from the lectin and classical pathways as it is constitutively active at low levels, a process called tickover [29,36]. During tickover, complement component C3 undergoes a conformation shift allowing for hydrolyzation of a thioester domain, forming C3(H_2_O), which can now bind Factor B [29,37]. The alternative pathway can also be activated by bacterial cell walls and endotoxins [29]. Once bound, Factor B is cleaved by Factor D releasing Ba and forming C3(H_2_O)Bb, a fluid-state initiation C3 convertase [29]. Factor D circulates in an inactive form called profactor D, which must be cleaved by MASP-3 to become enzymatically active [29]. The soluble C3 convertase cleaves C3 to C3a and C3b. C3a is an anaphylatoxin that is released into surrounding tissue and circulation, while the C3b product can covalently attach to a target surface and bind to Factor B [29]. Again, Factor B is cleaved by Factor D, but this time it is covalently attached to the target cell forming the alternative pathway C3 convertase, C3bBb [37]. The alternative pathway C3 convertase is relatively unstable, lasting only about 1.5 min [38]. However, when bound to the complement regulatory protein properdin, the complex becomes stable for about 15 min, which is the same length of time that the classical and lectin pathway convertase is stable [37,39].

#### 3.1.2. Response Expansion

During response expansion, all three pathways of activation converge at the formation of a C3 convertase bound to the surface of the target cell. For the classical and lectin pathways, the C3 convertase is C4bC2a. For the alternative pathway, the C3 convertase is C3bBb complexed with the regulatory protein properdin (C3bBbP). C3 convertase binds C3 and cleaves it into two fragments: C3a and C3b [29,33,40].

Cleavage of C3 and successive binding of Factor B is continually repeated by cross-pathway formation of C3 convertases in a process called amplification [36]. Through amplification, target cells can become opsonized by complement fragments resulting in their effective phagocytosis by immune cells [29]. Additionally, amplification leads to complement cascade progression through response expansion and eventually insertion of the membrane attack complex (MAC) intermembrane pore.

After C3 cleavage, the anaphylatoxin C3a is released into surrounding tissue and circulation where it carries out immunomodulatory functions [41,42,43]. C3b can bind to the target cell where it acts as an opsonization signal for phagocytes, or it can bind to the C3 convertase to form the C5 convertase (C4bC2aC3b or C3bBbC3b) [35]. C5 convertase functions similarly to its C3 counterpart, but it binds and cleaves C5 into the anaphylatoxin C5a, which is released into the surrounding tissue, and C5b, which is also a soluble molecule [41,42,43].

In a study of 30 infertile men, IgG and C3 were detected on abnormal basement membrane structures of seminiferous tubules in immune complex orchitis indicative of classical pathway activation of the complement system [44]. In the case of idiopathic male fertility, which makes up about two-thirds of male infertility cases, IgG and C3 were detected in 20–70% percent of testicular tissue from biopsies (*n* = 20–50) [44,45,46] and ASA were present in up to 6% of men that are referred to receive treatment for infertility [47]. Additionally, in a rabbit model of allergic orchitis, IgG and IgM ASA along with C3 deposits were detected in the testis [48]. Thus, activation of complement through C3 involvement has been detected, however no further studies have been conducted to investigate other complement components, such as C5 or MAC, in orchitis nor in the testis.

#### 3.1.3. Termination

C5b marks the beginning of MAC assembly. The C5b fragment remains soluble and binds C6, then C7 in solution [30,32,49]. Once C7 is bound, the C5b-7 complex can bind back to the target cell membrane and can also bind to C8. Binding to C8 can occur in solution or on the membrane, however it is only when C5b-7 is bound to the membrane that further MAC assembly can occur [41,42,43]. When C8 binds to C5b-7, the complex attaches with a much stronger affinity to the target cell and now can serve as an attachment point for C9, the pore fragment of MAC [50,51]. Anywhere from three to 18 (usually about 12) C9 molecules are recruited by the C5b-8 complex and inserted into the target cell membrane to form the MAC pore [29,51]. Insertion of multiple MACs into the target cell leads to cellular destruction by cytolysis and termination of the cascade. Unlike apoptosis, this is a highly inflammatory form of cell death that continues to attract inflammatory immune cells and further immune responses.

### 3.2. Functions of Complement

**Figure 3:**
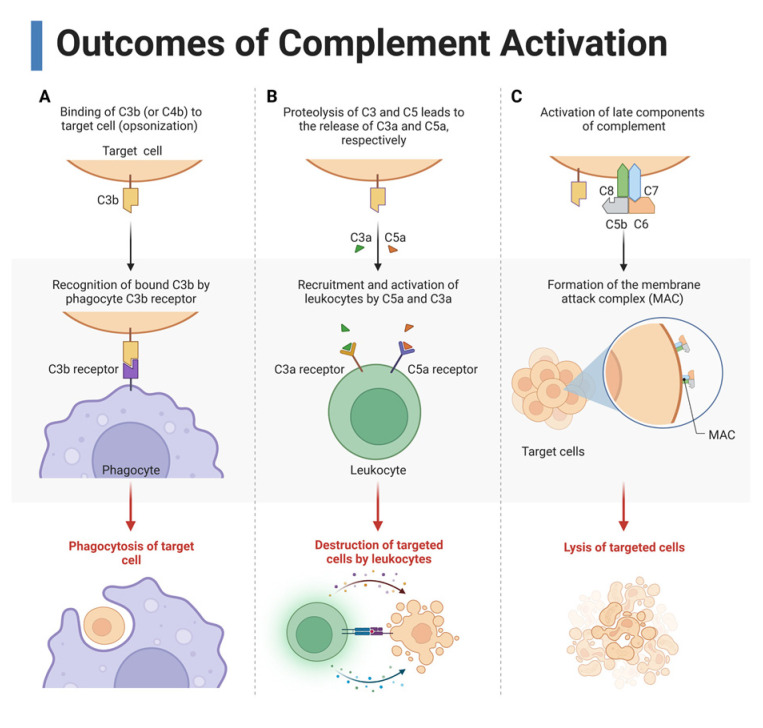
Three main outcomes of complement activation. (**A**) Complement cleavage products such as C3b opsonize the target cells, which allows for recognition by phagocytes for phagocytosis. (**B**) Cleavage of C3 and C5 form the anaphylatoxins C3a and C5a, respectively, which recruit and activate immune cells by binding to their receptors on leukocytes. (**C**) Late complement components C5, C6, C7, C8, and C9 form the membrane attack complex (MAC), a pore that causes cytolysis. This figure was created with BioRender, accessed on 1 December 2022.

Complement has three overarching functions in immunity: opsonization and phagocytosis, inflammation and immune modulation, and cytolysis (Figure 3).

#### 3.2.1. Three Main Outcomes of Complement Activation

As previously discussed, the complement system causes lysis of the target cell or pathogen by insertion of the MAC [35]. Many cleavage products are deposited on the target cell including C3b, C3c, C3d, C3f, C3dg, C4b, C4c, and C4d, which mark the cell for opsonization [29]. These fragments act as ligands to the complement receptors (CR) C1QBP, CD35, CD21, CR3, and CR4, which are expressed on immune cells including the phagocytes (Table 1) [52]. Ligation of CR allows for targeted and more efficient phagocytosis of the complemented cell and cellular debris [53]. Phagocytosed cell products are then presented for B and T cell activation.

Complement also modulates immune responses [49]. This occurs through immune cell interaction with anaphylatoxins, complement components, and complement inhibitory proteins, which will be discussed later. The anaphylatoxins are C3a and C5a, protein fragments formed from the cleavage of C3 and C5, respectively, that function to influence further immune response through induction of inflammation, recruitment of leukocytes, activation of immune cells, and modulation of the adaptive immune response [36,40]. Anaphylatoxins carry out these functions in an autocrine, paracrine, or systemic manner by binding to their respective receptors: C3a binds to C3a receptor (C3aR) and C5a binds to C5a receptor (C5aR1) or C5a receptor 2 (C5aR2) (Table 1) [49,54]. These receptors are expressed on leukocytes, endothelial cells, and epithelial cells [40,49]. Depending on which anaphylatoxin(s) are engaging which receptor(s) plays a large role in determining the type of response of the ligated cell.

**Table 1:** Receptors of complement components.

Complement Receptor	Ligands	Cellular Distribution	Role in Complement	Role in Male Reproduction	References
Type 1 complement receptor (CD35, CR1)	C3b, C4b, iC3b	T cells, B cells, macrophages, neutrophils, dendritic cells, eosinophils, erythrocytes	Cofactor for cleavage of C3b and C4b, opsonization, clears immune complexes	Associated with testicular interdigitating dendritic cell tumors; any role in reproduction is unknown	[52,55,56,57]
Type 2 complement receptor (CD21, CR2)	C3d, C3dg, iC3b	B cells, dendritic cells, epithelium	Coreceptor in B cell activation, sequesters antigens in lymphoid germinal centers	Unknown	[52,56]
Type 3 complement receptor (CR3, CD11b/CD18)	iC3b, ICAM-1	Macrophages, dendritic cells, neutrophils, natural killer cells	Opsonization, adhesion of leukocytes to endothelium	Unknown	[52,58,59]
Type 4 complement receptor (CR4, CD11c/CD18)	iC3b	Macrophages, dendritic cells, neutrophils, natural killer cells	Opsonization, adhesion of leukocytes to endothelium	Unknown	[52,58,59]
C1q binding protein (C1QBP, gC1q receptor, gC1qR)	C1q	T cells, B cells, monocytes, dendritic cells, neutrophils, endothelium	Opsonization, mitochondrial fitness, T cell activation and proliferation, regulates cellular cytotoxicity of B cells, chemotaxis induction	Unknown	[31,60,61]
Complement receptor of immunoglobulin family (CRIg)	C3b, iC3b	T cells, macrophages	Stabilizes Tregs, enhances Treg response, opsonization	Unknown	[62,63,64,65]
C3a receptor (C3aR)	C3a, C3a-DesArg	T cells, B cells, macrophages, dendritic cells, myeloid cells	Leukocyte chemotaxis and extravasation, T cell activation and proliferation, regulates cellular cytotoxicity	Unknown	[49,66]
C5a receptor 1 (C5aR)	C5a, C5a-DesArg	T cells, B cells, macrophages, dendritic cells, myeloid cells	Leukocyte chemotaxis and extravasation, T cell activation and proliferation, regulates cellular cytotoxicity	Unknown	[49,66]
C5a receptor 2 (C5L2)	C5a, C5a-DesArg	T cells, neutrophils, macrophages, dendritic cells	Negative regulator of C5aR, decoy receptor, proinflammatory and anti-inflammatory functions	Unknown	[67,68]

#### 3.2.2. Anaphylatoxin Functions

Anaphylatoxins are known to influence the adaptive immune response in three ways. First, anaphylatoxins encourage leukocyte chemotaxis and migration since immune cells migrate toward the C3a/C5a gradient [40]. Anaphylatoxins in circulation and tissue act as a trail of breadcrumbs, leading leukocytes to the site of complement activation [29]. Leukocytes follow the increasing concentration gradient of anaphylatoxins, particularly with the binding of C5a to C5aR. Anaphylatoxins are especially powerful chemoattractants for macrophages and T cells [53,69].

Second, anaphylatoxins can activate a pro-inflammatory response by engaging their receptors on endothelial and epithelial cells [49]. This binding encourages increased expression of leukocyte recruitment and chemotaxis molecules such as P-selectin and intercellular adhesion molecule-1 (ICAM-1), which allow for the tethering of circulating immune cells to blood vessel endothelium [70,71]. Furthermore, anaphylatoxins induce leukocytes to produce inflammatory cytokines such as tumor necrosis factor-α (TNF-α) and interferon-γ (IFN-γ) [49,72]. Anaphylatoxin receptor engagement leads to decreased expression of tight junctions such as claudin-5 and zona occludin 1, making the endothelium more permeable and more conducive for leukocyte extravasation into the affected tissue [73,74].

Third, when anaphylatoxins bind to their receptors on immune cells, they initiate a signaling cascade that leads to effector cell activation, survival, and proliferation [49]. In opposition, a decrease in anaphylatoxin receptor binding is a critical step in the generation of regulatory immune cells such as Tregs, which play an important role in suppressing the cytotoxic immune response [33,49,54,75,76]. Taken together, the role of anaphylatoxins in infection and graft rejection is to lead the immune cells to the site of complement activation and to create an environment where immune cells can more easily access the affected area. Nevertheless, anaphylatoxins and their effects have not yet been investigated in the testis. More information regarding the direct effect of anaphylatoxins on the immune cell responses will be provided later.

## 4. Complement Regulation

Due to the highly inflammatory nature of complement, tight regulation of the complement cascade by host cells is critical to prevent collateral damage, tissue injury, and autoimmunity. Complement can be inhibited at just about every step of the cascade through action of complement inhibitory proteins (Table 2). Complement inhibitors are either membrane-bound or soluble proteins that act to regulate the function of and prevent continued activation of complement. Membrane-bound complement inhibitory proteins inhibit complement components that directly interact with the cell expressing these inhibitors [50,77]. Soluble complement inhibitors can extend their regulatory effect further, affecting both the expressing cells and neighboring cells in a paracrine fashion [51]. Soluble complement inhibitors can also inhibit soluble complement components such as C5b, C5b-6, and C5b-7, thus thwarting insertion of the MAC [51].

### 4.1. Activation Pathway Inhibitors

Multiple complement inhibitors have been identified that target the classical, lectin, and alternative activation pathways to inhibit complement activation. Classical pathway inhibition is focused on the C1 complex, lectin pathway inhibition tends to occur by inhibiting MASP-1 and MASP-2, and alternative pathway inhibition focuses on Factor B. C1 inhibitor (C1INH, SERPING1), C1q binding protein (C1QBP), pentraxin (PTX3), and cartilage oligomeric matrix protein (COMP) are plasma soluble proteins that inhibit action of the C1 complex through inhibiting C1q, dissociating C1r/C1s, or preventing association of the C1 complex components [77,79,84,85,91,92,96].

Regarding the reproductive system, C1INH deficiency is associated with the development of hereditary angioedema, which can include presentation of testicular swelling [82,146]. C4BP is synthesized in the epididymis through an androgen-dependent mechanism, but its role in male reproduction is unknown [88]. In males, PTX3 has been detected in the male reproductive tract where it binds immotile spermatozoa and is correlated with the number of normal spermatozoa [97]. Furthermore, PTX3 is upregulated in oocytes prior to ovulation in mice and is important in female fertility [98].

SUSD4 is both a plasma soluble and membrane-bound inhibitor that prevents assembly of the classical and lectin C3 convertase by inhibiting cleavage of C2 [102]. SUSD4 is primarily expressed in the testis, brain, eye, and spinal cord—immune privileged organs [103]. Moreover, COMP and SUSD4 have also been shown to prevent activation of the lectin pathway by binding MBLs to prevent further activation [91,92]. MAP-1 and MAP-2 competitively bind to MBL to prevent MASP interaction [99,100,101]. Lastly, type 1 complement receptor 1 (CR1, CD35) acts as a membrane-bound inhibitor, preventing assembly of the C1 complex and association of MASP proteins with MBL, CL, or FCN. Additionally, CD35 acts with Factor I to inhibit the convertases, which will be discussed later [55,57].

Factor H is the only well-characterized inhibitors of the alternative pathway identified so far. Factor H is a plasma soluble protein that accelerates dissociation of Bb from C3b, and when acting as a cofactor for Factor I will cleave C3b to inactive fragments [93]. Thus, Factor H not only inhibits alternative pathway activation, but also amplification of the complement response. Additionally, Factor H is detected in the seminal plasma of pigs and the outer acrosome of sperm where it has been shown to protect sperm from complement-mediated destruction through reproductive tracts [95].

### 4.2. C3 and C5 Convertase Inhibitors

All three pathways of complement activation converge on the assembly of the C3 convertase, which functions to assemble the C5 convertase. As these are critical steps in complement function, inhibition of the convertases by different complement inhibitors is critical to shutting down undesired complement activation. Some of these complement inhibitory proteins have already been discussed: CD35 and Factor H—both are Factor I cofactors. Factor I is a plasma protein that circulates in an inactive state and requires a cofactor for activation. When activated, Factor I and its cofactor inhibit both the classical/lectin and alternative convertases by cleaving C4b and C3b into inactive fragments [124]. The Factor I cofactors are CD35, Factor H, complement factor H-related proteins 1–5 (CFHR1-5), C4 binding protein (C4BP), CUB and sushi domains protein 1 (CSMD1), and CD46 [124]. The plasma proteins CFHR1-5 also dissociate Bb from C3b and interact with C3b, C3dg, and iC3b to enhance opsonization [77,93]. VWF is a large serum protein that inactivates C3b by cleaving it to iC3b [130,131]. CD141 and PLG are both membrane-bound and serum inhibitors that inhibit complement response expansion by decaying the C3 and C5 convertases [104,105,125,126]. Concerning reproduction, CD141 is a marker for adenocarcinoma in the rete testis [107]. As recurrent miscarriage can be caused by defects in coagulation, a decrease of placental CD141 has been associated with this condition [106]. PLG is detected on oolemma and decreases the amount of sperm that penetrate into the oocyte [127,128].

CSMD1 also inhibits the terminal pathway by preventing binding of C7 to C5b-6 [102,122]. CD46, also known as membrane cofactor protein (MCP), is a membrane bound inhibitor that also plays an important role in the regulation of T cells, which will be discussed later. Furthermore, CSMD1 and CD46 have been shown to have roles related to fertility [123,147]. CSMD1 has been detected at the Sertoli cell-Sertoli cell and Sertoli cell-spermatid interfaces, and CSMD1 knockout male mice have decreased fertility, increased testicular C3 deposition, and testicular degradation [123]. On sperm and spermatids, CD46 is located solely on the acrosomal membrane and is believed to be important in stabilizing the acrosome reaction during egg-sperm fusion [108,109,110].

The last two convertase inhibitors are membrane-bound complement receptor of the immunoglobulin superfamily (CRIg) and CD55. CRIg is a membrane bound inhibitor and the only complement receptor with immunoglobulin domains that converts C3b and iC3b and also acts as a negative regulator of T cell activation and proliferation [65,120]. The glycophosphatidylinositol (GPI)-anchored CD55, also known as decay-accelerating factor (DAF), accelerates the decay of the convertases by dissociating C2a and Bb from C4b and C3b, respectively, and plays a role in regulating T cell tolerance [117,118]. CD55 has been detected on the inner acrosomal membrane of sperm where it likely is important in protecting sperm from female complement-mediated destruction and may play other roles in reproduction [115], which should be investigated further.

### 4.3. Terminal Pathway Inhibitors

Even if complement activation proceeds through the convertases, the various components of the terminal pathway can be inhibited through other complement inhibitors. Clusterin is a membrane-bound and plasma soluble inhibitor expressed at significantly high levels in seminal plasma [137]. Clusterin is thought to influence maturity processes of sperm [135] and to prevent male stress proteins from aggregating allowing them to be endocytosed by dendritic cells [138]. In this manner, clusterin may be creating a tolerogenic environment within the semen to allow sperm to survive in the female reproductive tract. Regarding complement, clusterin inhibits MAC assembly by binding the terminal complement components to prevent insertion of the MAC [50,136,137]. Clusterin can act with plasma-soluble vitronectin to interact with free C5b-7, C5b-8, and C5b-9 to form the soluble terminal complement complex (TCC, or sC5b-9) [50,136,137]. On its own, vitronectin inhibits C8 binding, but also maintains vascular homeostasis and promotes cell adhesion and migration processes involved with tissue repair [139]. In reproduction, vitronectin plays a role in sperm aggregation and adherence to the oocyte [141]. Lastly, CD59 is a GPI-anchored membrane protein that acts as a suicide inhibitor of the terminal pathway by irreversibly binding to C8 to block C9 recruitment [133,148]. Like the other GPI-anchored complement inhibitor CD55, CD59 is also expressed on the inner acrosomal membrane where it may protect sperm from female complement [115] and has been shown to correlate positively with testicular androgen synthesis [134].

### 4.4. Anaphylatoxin Inhibitors

In addition to inhibitors of complement cascade components, anaphylatoxins can also be inhibited by complement inhibitory proteins called carboxypeptidases (CPX). The specific CPX that inhibit anaphylatoxins are CPA, CPB2, CPN1, CPN2, and CPR [142,143,144,145]. These are primarily synthesized by hepatocytes and are plasma soluble circulating proteins [142]. CPX proteins remove the carboxy terminus from C3a and C5a, converting them to C3a desArg and C5a desArg, respectively. Though C3a desArg loses its ability to bind C3aR, C5a desArg can still engage C5aR, but at 10–15-fold lower affinity than C3a and C5a [41,149]. Interestingly, C5a desArg can bind with high affinity to C5L2, which may lead to anti-inflammatory effects [67,68]. In addition to inhibiting the action of anaphylatoxins, CPX also reduce degradation of the extracellular matrix, inhibit cellular migration of leukocytes, and inhibit fibrinolysis [142,144].

Overall, the complement inhibitors function to keep complement activation in check to prevent detrimental inflammation and overactivation of the immune system. Some of the inhibitors may play important roles in reproduction and immune privilege. Furthermore, CD35, CD46, CD55, and CD59 even play roles in T cell activation and differentiation [117,118,150]. Along with CRs and anaphylatoxins, complement inhibitory proteins can shape the course of innate and adaptive immune cell responses.

## 5. Complement Anaphylatoxin Modulation of Immune Cells

Complement fragments and regulatory proteins influence the action of immune cells to modulate the course of the immune response to foreign antigens such as pathogens, immunogenic germ cells, and grafted cells [50,151]. Although complement interacts with all the immune cells, this review will focus on its interaction with antigen presenting cells (APC) and T cells.

### 5.1. Antigen Presenting Cells

APC are phagocytes that ingest pathogens, cells, and cellular debris, and then present the antigens on their cell surface for T cell recognition. T cells are lymphocytic immune cells that mediate cellular immune responses after recognition of antigens, either through activating other immune cells or by direct cytotoxic killing of infected cells. If T cells recognize the antigen as foreign, then they initiate further immune response by releasing cytokines to recruit and activate leukocytes to destroy any other cells which express that antigen [152].

The most abundant immune cell population in the testis are testicular macrophages [153]. Macrophages are innate, large mononuclear phagocytes that function to destroy pathogens and clear apoptotic cells [154]. Macrophages have two main phenotypes: M1 and M2. M1 macrophages produce and secrete proinflammatory cytokines to destroy microbes while M2 macrophages are associated with anti-inflammatory effects and tissue repair [155,156]. The M1 macrophage phenotype is most implicated in the case of graft rejection and inflammatory autoimmune diseases [155]. They are the primary cellular mediators of robust xenograft rejection, which can occur directly by the macrophage itself, or indirectly through macrophage activation of T cells [157,158,159,160]. On the other hand, anti-inflammatory M2 macrophages are associated with tolerogenic environments [155]. In fact, testicular macrophages, the primary immune cell type within the testis, have an M2 or M2-like phenotype conducive to immune-privilege [161,162,163].

Testicular macrophages are important in establishing immune tolerance to germ cell antigens, infection response, and control of inflammation [164]. Because of these roles, testicular macrophages contribute to immune regulation in the testis. However, in rats with experimental autoimmune orchitis, testicular macrophages participate in disease pathogenesis through production of proinflammatory cytokines and activation of T cells, so proper balance of M1 and M2 phenotypes is critical [165]. Since macrophages are so abundant in the testis, it makes sense that they are among the first APC to infiltrate the inflammatory testicular environment of autoimmune orchitis [166]. Interestingly, they are also among the first APC that infiltrate into grafts [166].

Another phagocytic APC, dendritic cells, are the most potent activators of T cells and the only known activators of naïve T cells. Dendritic cell presence in an autoimmune and rejecting graft can lead to a strong T cell response [160,167]. Conversely, under the right circumstances dendritic cells can act in a tolerogenic fashion and can generate an immune-protective Treg response [168]. Though dendritic cells make up a small percentage of testicular immune cells, it is these tolerogenic or immature dendritic cells that are the primary dendritic cell population in the testis [162,169].

Interestingly, the complement anaphylatoxin receptors C3aR, C5aR1, and C5aR2 have been shown to affect phagocyte response and function during episodes of complement activation. Ligation of anaphylatoxin receptors induces chemotaxis of phagocytes toward an increasing anaphylatoxin gradient. This signaling pathway causes immune cells such as macrophages and T cells to follow the increasing C3a/C5a gradient toward the site of complement activation [170]. Anaphylatoxin receptor interaction enhances phagocytosis and leads to the production and release of pro-inflammatory cytokines such as IL-6, TNF-α, and IL-1β [171]. Regarding high-inflammatory cells such as neutrophils, the anaphylatoxin signaling pathway activates neutrophils to degranulate and undergo respiratory burst, and C5aR1 inhibits their apoptosis, thus increasing their capacity to destroy grafts [171,172,173].

Ultimately, anaphylatoxin receptor ligation on APC leads to cell migration toward the graft, cell adhesion and extravasation into the graft, enhanced phagocytosis, release of proinflammatory factors, and overall increases efficiency of phagocytes to destroy grafted cells [170]. In the absence of C3aR or C5aR1 ligation however, the tolerogenic APC phenotype become more dominant, thus encouraging the induction of immune regulatory lymphocytes [169,174]. The role of CD46 and other complement regulators on APC outside of complement regulation has yet to be fully elucidated, but preliminary studies indicate that CD46 may play a role in the polarization of innate immune cells [175].

### 5.2. T Cells

The primary purpose of phagocytes acting as APC is to activate T cells [169]. T cells are most rigorously studied pertaining to their role in autoimmunity, cancer, and transplant survival outcomes. T cells are divided into the following subsets: helper T cells (Th, CD4^+^CD25^lo^Foxp3^lo^), cytotoxic T cells (CTLs, CD8^+^CD25^lo^Foxp3^lo^), and regulatory T cells (Tregs, CD4^+^CD25^hi^Foxp3^hi^ and/or CD8^+^CD25^hi^Foxp3^hi^) [16,176,177,178,179,180].

Th function in the cellular adaptive immune response by priming immune cells including phagocytes, B cells (antibody-producing cells), and CTLs to target cells whose major histocompatibility complex (MHC) express specific antigens. Th1 cells release proinflammatory cytokines such as IL-1 and IL-17 to activate the cytotoxic immune functions of macrophages and CTLs [154,181,182]. IL-1 serves to recruit leukocytes, induce expression of cell adhesion molecules, and promote leukocyte extravasation; while IL-17 activates and mobilizes inflammatory immune cells [182].

After activation by Th, CTLs directly kill target cells and specialize in the destruction of viral-infected cells and tumor cells. CTLs are a critical component of the cellular immune response against MHC-mismatched allografts in acute rejection [177,180]. CTLs directly lyse target cells through granule exocytosis of perforin and granzyme B [180]. Perforin forms an intermembrane pore that allows granzyme B, a pro-apoptotic mediator, to enter the cell and trigger cell death [183]. CTLs also recruit immune cells to the area through the release of proinflammatory cytokines [178,180]. Overall, activation of CTLs against a cell leads to cytotoxicity.

In contrast to Th and CTLs, Tregs suppress and contract the cytotoxic immune response, and they accomplish this through multiple mechanisms. In particular, Tregs release anti-inflammatory mediators such as TGF-β and IL-10, which suppress function and proliferation of Th1 cells [184]. They also express the IL-2 receptor CD25, which has a high affinity for IL-2 binding and sequesters IL-2, a growth factor for T cell survival and proliferation [184]. Acting together, Treg expression of these factors prevent Th1 cell expansion, suppresses inflammation, and contracts the immune response [184].

High levels of anaphylatoxins binding to their receptor on T cells activates a signaling cascade that encourages high-affinity binding of the MHC-TCR (T cell receptor) complex. This leads to the generation, activation, and expansion of Th1 cells and CTLs [49,185]. Specifically, anaphylatoxin-receptor engagement on T cells decreases expression of the pro-apoptotic Fas receptor and increases expression of the anti-apoptotic molecule B cell lymphoma-2 (Bcl-2) [49]. The anaphylatoxin signaling cascade suppresses production of IL-4, a cytokine that stimulates T helper 2 cells (Th2, Th subset associated with wound healing and tissue repair) differentiation, and inhibits production of TGF-β, which is important in Treg generation. Moreover, when anaphylatoxins bind their receptors on differentiated Tregs, their suppressive functions are downregulated [54]. This effect was confirmed when C3aR/C5aR1 receptors were knocked down and Treg suppression was enhanced in the presence of anaphylatoxins [54]. Thus, C3aR/C5aR1 ligation on CD4^+^ T cells promotes differentiation of Th1 cells and inhibits generation of anti-inflammatory Tregs. In the case of pathogen infiltration, this is a desired result, as these proinflammatory CD4^+^ T cells develop an immune response that is necessary to clear the infection, but in autoimmunity and transplantation this response leads to the destruction of host cells and grafts.

Ligation of C5aR1 on T helper 1 cells (Th1, Th subset associated with mounting pro-inflammatory response) induces the production of reactive oxygen species (ROS) and development of the inflammasome, which is important in the expression of IL-1β [171,186]. C5aR1 signaling on CTLs induces a stronger proliferative response and increases expression of perforin and proinflammatory cytokines [187]. Overall, anaphylatoxin receptor signaling leads to enhancement of pro-inflammatory Th1 cell and CTL responses. Conversely, C5aR2 tends to act antagonistically to C5aR1 by sequestering C5a and preventing further signaling through C5aR1, and other functions of this receptor are currently under investigation [67,68].

## 6. Sertoli Cell Regulation of Complement

Activation of the complement cascade inevitably leads to destruction of the target cell [49,188,189], which is why Sertoli cell survival of complement activation is so unique. In vitro complement cytotoxicity assay experiments have shown that control cells (pig endothelial cells and pig islets) are killed by robust human and rabbit complement, while mouse and pig Sertoli cells survive [25,190,191]. Pig Sertoli cells and control cells (pig endothelial cells) cultured on chamber slides were exposed to human serum, and human serum + rabbit complement and immunostained for deposition of the complement proteins C4b, Bb, C3b, and MAC [190]. C4b and C3b were deposited on both the controls and the Sertoli cells, while Bb and MAC were only observed significantly deposited on the controls [190]. These results coincide with cell death (endothelial cells) and cell survival (Sertoli cells) of complement and imply that Sertoli cells are inhibiting complement before MAC deposition. In vivo data agreed with the in vitro results as basically no complement deposition of C4, C3, or MAC was observed on mouse Sertoli cell allografts or pig Sertoli cell xenografts through day 20 post-transplantation [25,191]. One mechanism Sertoli cells could be using to inhibit the complement cascade is expression of complement inhibitory proteins.

**Figure 4:**
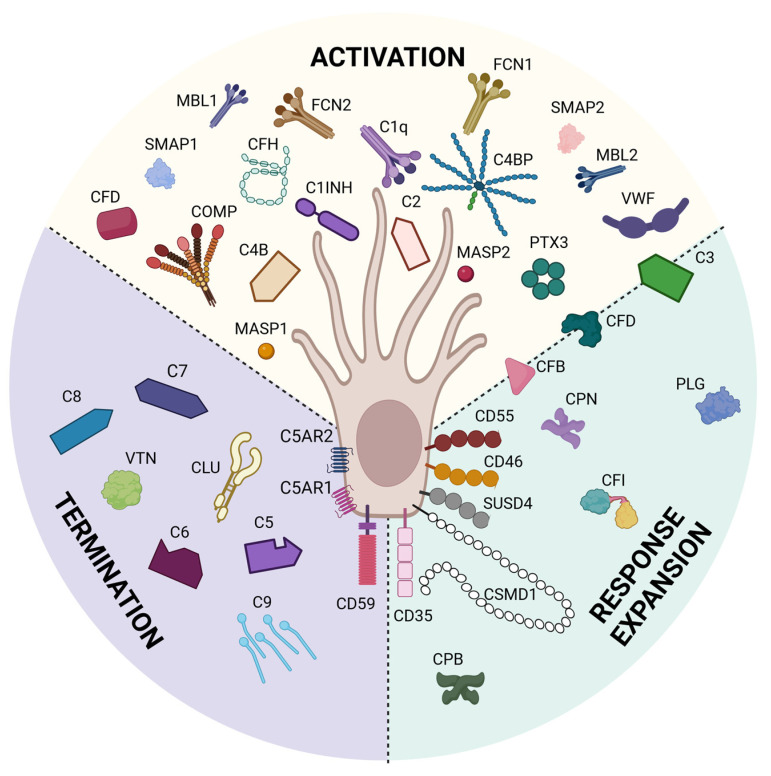
Sertoli cells express complement proteins. Bioinformatic analyses have identified 21 complement inhibitors, 25 complement factors, and 3 complement receptors expressed by Sertoli cells. These inhibitors block complement throughout the whole cascade from activation (yellow), response expansion (green), and termination (purple). This figure was created with BioRender, accessed on 1 December 2022.

We previously have shown through bioinformatic analyses that mouse Sertoli cells express 14 complement inhibitor genes [191] and pig Sertoli cells express 21 complement inhibitor genes [192] that inhibit all throughout the complement cascade (Figure 4). qPCR analyses have shown that pig Sertoli cells express C1INH, CD35, CD46, CD55, COMP, CPN2, CSMD1, and PTX3 genes at significantly elevated levels as compared to pig islet controls, which are killed by complement [25,192]. ELISA of Sertoli cell conditioned media confirmed mouse Sertoli cell secretion of C1INH, C1QBP, and COMP [191] and confirmed pig Sertoli cell secretion of CPN2 and PTX3, also significantly elevated above pig islet controls [192]. Protein analyses of pig Sertoli cell complement inhibitors by Western blot revealed no change in CLU, but significantly higher expression of CD46 by pig Sertoli cells as compared to pig islets, and that CD55 expression was only detectable in Sertoli cells [25]. These studies demonstrate that Sertoli cells express many complement inhibitory proteins that inhibit critical points throughout the complement cytolytic cascade, some of which are involved in T cell activation and differentiation. Future study is needed to determine the importance of these inhibitors individually and together in Sertoli cell survival and germ cell protection.

Additional RNA sequencing analysis determined that pig Sertoli cells express nearly all complement cascade genes (only C1r was not detected) and three complement receptor genes (CD35, C5aR1, and C5aR2) [192]. The reason behind this complement expression remains understudied, however it is possible that Sertoli cells express complement cascade components to provide an antimicrobial response in the seminiferous tubules if infected. However, as complement components increasingly have been shown to regulate part of immune function (discussed previously), this role may be more complicated and is worthy of future study.

As previously discussed, complement can affect immune cell recruitment and activation. Interestingly, in the aforementioned pig Sertoli cell xenografts, a delay of macrophage migration into the surviving Sertoli cell grafts was observed as compared to the rejecting pig islet xenografts at days four and six post-transplantation [16]. Since the presence of early macrophage infiltrate within the control xenografts is indicative of transplant rejection, this indicates that Sertoli cells are impeding macrophage recruitment.

CD4^+^ and CD8^+^ T cell infiltrate was also analyzed in these xenografts. After flow cytometry and immunohistochemical (IHC) analyses of CD4^+^ T cells, no significant difference in migration between the two types of grafts was measured at early timepoints [16]. CD4^+^ Treg infiltrate within the xenografts was further analyzed to determine if any of the CD4^+^ T cells were Tregs, which are associated with graft survival. Both IHC (CD4^+^Foxp3^+^ markers) and flow cytometry (CD4^+^CD25^+^Foxp3^+^ markers) for Tregs indicated that 30% of the CD4^+^ T cell infiltrate in Sertoli cells grafts was Tregs and that this number was significantly higher at day four post-transplantation in Sertoli cell grafts [16]. In fact, the Treg/Th ratio at days four and six were 0.85 and 0.48 in Sertoli cell xenografts, while the Treg/Th ratio in control islet xenografts at the same time points were significantly lower, at 0 and 0.39 [16]. In effect, Th cells were the only measurable CD4^+^ T cells in the control xenografts at day four, as no Tregs were detectable in the control grafts until day six post-transplantation [16].

CD8^+^ T cell infiltrate was also measured in these xenografts. Sertoli cell xenografts had significantly more CD8^+^ T cells at day four post-transplantation than control grafts, however at day six post-transplantation there was no significant difference in CD8^+^ T cell numbers between controls and Sertoli cell grafts [16]. The grafts were also analyzed for CD8^+^ Tregs by flow cytometry (CD8^+^CD25^+^Foxp3^+^ markers)and IHC (CD8^+^Foxp3^+^ markers) [16]. At day four post-transplantation, CD8^+^ Tregs were only observed in Sertoli cell grafts and, at day six post-transplantation, CD8^+^ Treg infiltrate in Sertoli cell grafts with significantly higher numbers compared to control islet grafts [16]. The presence of so many Tregs at day four and the continued presence of Tregs at day six post-transplantation in the Sertoli cell grafts implies that their survival as xenografts is associated with an immune suppressive environment [16].

Overall, this study demonstrates a delayed infiltration of immune effector cells and increased infiltration of Tregs into Sertoli cell xenografts by day six post-transplantation [16]. As complement anaphylatoxins are important in effector immune cell activation and recruitment, it is possible that the expression of so many different complement inhibitors by Sertoli cells are restraining the formation of anaphylatoxins, which in turn is decreasing effector infiltrate and encouraging Treg development. The information discussed clearly shows that Sertoli cells regulate immune responses and complement in transplantation models, and we speculate that Sertoli cells also perform this for germ cell protection. Complement has not really been studied in the testes, except briefly in the context of autoimmune orchitis, where anti-sperm antibodies bind to germ cells and have the potential to activate complement. Thus, one role Sertoli cells could be performing is to regulate complement to protect germ cells from an undesired immune response [26,27].

## 7. Discussion

Sertoli cells create an immunoregulatory environment within the testis to protect immunogenic germ cells. Sertoli cells are also able to accomplish this within immunogenic grafts, and the mechanisms behind this are currently under investigation. The focus of this review was Sertoli cell inhibition and modulation of complement in immunoregulatory environments. Complement components, anaphylatoxins, receptors, and inhibitory proteins can modulate phagocytes, T cells, and B cells to generate either a damaging pro-inflammatory cytotoxic response, or a tolerant anti-inflammatory immune suppressive response [75,90,118,193]. When complement becomes dysregulated, it leads to destruction of host cells, germ cells, and transplants [194]. Thus, the proper regulation of complement is imperative in immune privilege.

In summary, Sertoli cells have been shown to survive robust xenogeneic human complement and rabbit complement in vitro, and the complement cascade termination component MAC was not detected on Sertoli cell allografts or xenografts [25,190,191]. Gene expression of 21 different complement inhibitors has been identified so far in Sertoli cells, eight have elevated gene expression (C1INH, CD35, CD46, CD55, COMP, CPN2, CSMD1, and PTX3) and four of these (CD46, CD55, CPN2, and PTX3) had elevated protein expression as compared to islets, which are killed by complement [25,191,192]. Furthermore, Sertoli cell xenografts have significantly elevated Treg infiltrate and decreased macrophage, Th, and CTL presence at days four and six post-transplantation as compared to control xenografts [16,49]. This could potentially be due to decreased anaphylatoxin levels resultant from Sertoli cell inhibition of complement and should be investigated. As Tregs are associated with graft survival, while Th cells, CTLs, and macrophages are associated with graft destruction, this is indicative of an immune protective environment.

**Figure 5:**
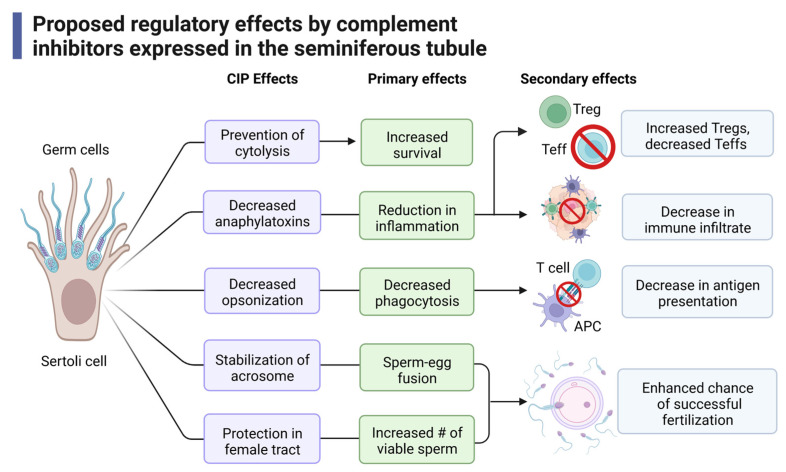
Proposed mechanisms of immune regulation by complement inhibitors expressed in the seminiferous tubule. Expression of complement inhibitors by Sertoli cells and germ cells play many different and distinct roles including direct inhibition of the complement system, modulation of immune cell activation, fertility, and fertilization. CIP: complement inhibitory proteins. This figure was made with BioRender, accessed on 1 December 2022.

Taken together, we propose that Sertoli cells express complement inhibitors to regulate the immune response in establishing immune protective environments and that this may be accomplished through the following mechanisms (Figure 5). Complement inhibitors prevent progression through the complement cascade, preventing cytolysis and increasing survival of the cells [195]. This inhibition decreases levels of anaphylatoxins produced to directly reduce inflammation. Furthermore, a reduction in anaphylatoxins also reduces signaling through C3aR and C5aR, which decreases effector T cell (Teff, Th, and CTL) activation while allowing for the induction of Tregs that help to sustain an immune suppressed environment [49,75,196]. Along these lines, immune cell recruitment is also decreased [42,53,170]. Opsonization markers are also diminished, which reduces phagocytosis and antigen presentation.

The acrosome of sperm is a vesicle that is secreted during the acrosome reaction. The acrosome reaction, which is critical in sperm–egg fusion, is when the outer membrane of the acrosome fuses with the plasma membrane of the oocyte [197]. The complement inhibitor CD46 has been shown to stabilize the acrosome to prevent premature and spontaneous acrosomal reactions, thus allowing for more effective sperm–egg fusion [197,198]. Other complement inhibitors (CD55, CD59, CLU) provide protection of complement within the female reproductive tract, increasing the number of viable sperm (Figure 5) [109,135,147]. Together, these functions allow for an enhanced chance of successful fertilization.

Though these studies have identified many novel and important complement regulatory functions of Sertoli cells in immune privilege, most of this work has been performed in transplantation studies. Heretofore, there is a knowledge gap in the understanding of the impact and importance of complement regulation in the testis and in reproduction. Elucidating the function(s) of complement in immune protection and reproduction may shed some much-needed light for males struggling with infertility.

## 8. Conclusions

Sertoli cells express a variety of complement inhibitory proteins that block just about every point of the complement cascade thus preventing MAC-mediated cytolysis, opsonization, and anaphylatoxin production. Not only does the expression of complement inhibitors protect Sertoli cells, but it also prevents the development of inflammation which reduces effector immune cell infiltrate while potentially allowing for the generation of regulatory cells such as Tregs, which aid in survival. As more studies are conducted in Sertoli cell regulation of complement, expression of more complement inhibitors, complement receptors, and even complement components will be revealed that may continue to shed light on the development of an immune-protective environment both within the testes and within grafts. Overall, understanding Sertoli cell regulation of the complement system has many applications in reproduction, autoimmunity, inflammation, and transplantation.

## Figures and Tables

**Table 2:** Complement inhibitory proteins.

Complement Inhibitor	Target	Distribution	Role in Complement	Role in Male Reproduction	References
**Activation pathway inhibitors**					
C1INH (C1 inhibitor, SERPING1)	C1r, C1s	Serum protein (0.21–0.341 μg/mL)	Dissociates C1r and C1s from C1q, controls vascular permeability	Deficiency causes hereditary angioedema, which may lead to testicular swelling	[78,79,80,81,82]
C1QBP (C1q inhibitor, C1INH, gC1qR,)	C1q	Serum protein (38 μg/mL)	Binds C1q to prevent further C1 complex assembly	Unknown	[83,84,85,86]
C4BP * (C4 binding protein, proline rich protein, PRP)	C4b	Serum protein (300 μg/mL)	Degrades C4b with Factor I, prevents assembly of classical and lectin pathway C3 convertases	Does not affect fertility, but may be involved in male reproduction, but the role is unknown	[87,88]
CD35 * (Type 1 complement receptor, CR1)	C1q, MBL, C3b, C4b, iC3b	Membrane protein	Associates with C1q or MBL to prevent activation, degrades C3b to iC3b and C3f, degrades iC3b to C3c and C3d,g, acts in antigen presentation, may generate Tregs	Associated with testicular interdigitating dendritic cell tumors; any role in reproduction is unknown	[57,88,89]
COMP (Cartilage oligomeric matrix protein)	C1q, MBL, C3	Serum protein (4–10 μg/mL)	Associates with C1q, MBL, and C3 to prevent further classical or lectin pathway activation	Unknown	[90,91,92]
Factor H (Reconnectin)	Bb, C3b	Serum protein (135–250 μg/mL)	Degrades C3b with Factor I, accelerates dissociation of Bb from C3 convertase	Detected in seminal plasma, protects sperm from complement-mediated destruction in male and female reproductive tracts	[93,94,95]
PTX3 (Pentraxin-related protein, TNF-inducible gene 14 protein)	C1q	Serum protein (0.002 μg/mL)	Binds C1q to prevent further C1 complex assembly	Detected in seminal plasma, associates with immotile spermatozoa, correlated with normal spermatozoa; important in female fertility	[96,97,98]
SMAP-1/SMAP-2 (MBL/ficolin/CL-associated protein, MAP44, MAP144)	MASP-1/2	Serum protein (1.4 μg/mL)	Prevents MASP-1/2/3 from binding MBL	Unknown	[99,100,101]
SUSD4 (Sushi-domain containing protein 4)	C1q, MBL	Membrane protein	Associates with C1q or MBL to prevent cleavage of C2	Unknown	[90,102,103]
**C3 and C5 convertase inhibitors**					
CD141 (thrombomodulin, granulocyte macrophage-colony stimulating factor, GM-CSF)	C3, C3a, C3b, C4b, C5a	Membrane and serum protein (0.003–0.3 μg/mL)	Causes decay of convertases; inhibits anaphylatoxins	Marker for adenocarcinoma of the rete testis; deficiency may contribute to recurrent miscarriage	[104,105,106,107]
CD46 * (Membrane cofactor protein, MCP)	C3b, C4b	Membrane protein	Degrades C3b/C4b with Factor I, dissociates C3b/C4b from C3/C5 convertases, important for Th1 response, contracts Th1 cells to Tregs	Located on the acrosomal membrane; Important in stabilizing the acrosome reaction during egg-sperm fusion	[108,109,110,111,112,113,114]
CD55 (Decay accelerating factor, DAF)	C3, C3b, C5, C5b	Membrane and serum protein (0.004–4 μg/mL)	Accelerates decay of C3 and C5 convertases, inhibits C3 and C5 cleavage, regulates T cell tolerance	Detected on the inner acrosomal membrane of sperm; May protect sperm from female complement-mediated destruction; May have other roles in reproduction	[115,116,117,118]
CFHR1-5 * (Complement factor H related proteins)	C3b, C3d,g, iC3b	Serum protein (6–100 μg/mL)	Degrades C3b with Factor I, binds C3b, interacts with C3d,g and iC3b, binds heparin	Unknown	[119]
CRIg (Complement receptor of immunoglobulin, V-set Ig domain-containing 4)	C3b, iC3b	Membrane protein	Associates with C3b to inhibit alternative pathway, binds iC3b, negatively regulates T cell activation	Unknown	[62,64,65,120]
CSMD1 * (CUB/sushi domains protein 1)	C3b, C7	Membrane bound	Degrades C3b with Factor I, inhibits binding of C7 to C5b-6	Located at the interface of germ cells and somatic cells; Mutations in this protein are associated with increased C3 deposition in testis and infertility in males and females	[121,122,123]
Factor I (ARMDI3, AHUS3)	C3b, C4b	Serum protein (35 μg/mL)	Degrades C3b and C4b, requires cofactor for activation	Unknown	[124]
PLG (plasminogen)	C3, C5	Membrane and serum protein (75 μg/mL)	Causes decay of convertases	PLG located on female germ cells reduces penetrant sperm; PLG activators are associated with testicular restructuring in development	[125,126,127,128,129]
VWF (von Willebrand Factor)	C3b	Serum protein (50–200 μg/mL)	Cleaves C3b to iC3b	Unknown role in male reproduction; Defective VWF increase miscarriage rate	[130,131,132]
**Terminal pathway inhibitors**					
CD59 (Protectin)	C8	Membrane protein	Interacts with C8 to prevent C9 recruitment, alternative ligand for CD2, activates T cells	Expressed on the inner acrosomal membrane; Protects sperm from female complement; Correlated with testicular androgen synthesis	[90,115,118,133,134]
Clusterin (CLU, sulphated glycoprotein-2, apolipoprotein J)	C6, C7, C8, C9	Membrane and serum protein (50 μg/mL)	Binds C6, C7, C8, and/or C9 to inhibit MAC assembly, forms soluble complexes with C5b-9, acts with vitronectin to prevent MAC insertion into membrane	Expressed at high levels in seminal plasma; May influence maturity processes of sperm; Prevent male stress proteins from aggregating; May contribute to sperm survival in the female reproductive tract	[135,136,137,138]
Vitronectin (VTN, Serum spreading protein, S-protein, plasminogen activator inhibitor-1 binding protein, epibolin)	C7	Plasma protein (200–400 μg/mL)	Interacts with C5b-7 to prevent C8 binding, acts with clusterin to prevent MAC insertion into membrane, forms soluble complexes with C5b-9, promotes cell adhesion and migration in tissue repair	Released within spermatozoon after acrosome reaction; Promotes sperm aggregation and sperm adherence to oocyte	[136,139,140,141]
**Anaphylatoxin inhibitors**					
CPB/CPN (Carboxypeptidases B, N)	C3a, C5a	Serum protein (30 μg/mL)	Degrades C3a/C5a to C3a/C5a desArg, inhibits migration of leukocytes, inhibits fibrinolysis	Unknown	[142,143,144,145]

* Factor I cofactor.

## Abbreviations

APC: Antigen presenting cell. C1INH: C1 inhibitor, SERPING1. C1QBP: C1q binding protein, C1q inhibitor, C1qI. C3aR: C3a receptor. C4BP: C4 binding protein. C5aR: C5a receptor 1. C5aR2: C5a receptor 2.CD21: Type 2 complement receptor, CR2. CD35: Type 1 complement receptor, CR1. CD46: Membrane cofactor protein, MCP. CD55: Decay-accelerating factor, DAF. CFHR: Complement factor H-related protein. CIP: Complement inhibitory protein. CL: Collectin. COMP: Cartilage oligomeric matrix protein. CPB: Carboxypeptidase B. CPN: Carboxypeptidase N. CR: Complement receptor. CRIg: Complement receptor of the immunoglobulin family. CSMD1: CUB and sushi domain protein 1. CTL: CD8+ cytotoxic T cell. FCN: Ficolin. GPI: Glycophosphatidylinositol. IDO: Indoleamine-2, 3-dioxygenase. IFN: Interferon. IHC: Immunohistochemistry. IL: Interleukin. MAC: Membrane attack complex. MASP: MBL-associated serine proteases. MBL: Mannose-binding lectin. MHC: Major histocompatibility complex. PD-L: Programmed death ligand. PLG: Plasminogen. PTX3: Pentraxin 3. SMAP1: Small MBL-associated protein 1. SMAP2: Small MBL-associated protein 2. SUSD4: Sushi-domain containing protein 4. TCR: T cell receptor. Teff: Effector T cell (Th and CTL). Th: CD4+ helper T cell. TGF-β: Transforming growth factor beta. THBS: Thrombospondin. TNF: Tumor necrosis factor. Treg: Regulatory T cell. VTN: Vitronectin. VWF: von Willebrand factor.
